# Effects of radiant exposure values using second and third generation light curing units on the degree of conversion of a lucirin-based resin composite

**DOI:** 10.1590/1678-77572016-0388

**Published:** 2017

**Authors:** Kelly Antonieta Oliveira Rodrigues de Faria CARDOSO, Driellen Christine ZARPELLON, Camila Ferreira Leite MADRUGA, José Augusto RODRIGUES, Cesar Augusto Galvão ARRAIS

**Affiliations:** 1 Universidade Guarulhos, Centro de Pós Graduação e Pesquisa, Guarulhos, SP, Brasil.; 2 Universidade Estadual de Ponta Grossa, Departamento de Odontologia, Ponta Grossa, PR, Brasil.

**Keywords:** Composite resins, Dental photoinitiators, Dental curing lights

## Abstract

Alternative photoinitiators with different absorption wavelengths have been used in resin composites (RCs), so it is crucial to evaluate the effectiveness of light-curing units (LCUs) on these products.

**Objective:**

Using Fourier transform infrared analysis (FTIR) *in vitro*, the effects of varying radiant exposure (RE) values generated by second and third generation LED LCUs on the degree of conversion (DC) and maximum rate of polymerization (Rp^max^) of an experimental Lucirin TPO-based RC were evaluated.

**Material and Methods:**

1 mm or 2 mm thick silicon molds were positioned on a horizontal attenuated total reflectance (ATR) unit attached to an infrared spectroscope. The RC was inserted into the molds and exposed to varying REs (18, 36 and 56 J/cm^2^) using second (Radii Plus, SDI) and third generation LED LCUs (Bluephase G2/Ivoclar Vivadent) or a quartz tungsten based LCU (Optilux 501/SDS Kerr). FTIR spectra (n=7) were recorded for 10 min (1 spectrum/s, 16 scans/spectrum, resolution 4 cm^-1^) immediately after their application to the ATR. The DC was calculated using standard techniques for observing changes in aliphatic to aromatic peak ratios both prior to, and 10 min after curing, as well as during each 1 second interval. DC and Rp^max^ data were analyzed using 3-way ANOVA and Tukey’s *post-hoc* test (p=0.05).

**Results:**

No significant difference in DC or Rpmax was observed between the 1 mm or 2 mm thick specimens when RE values were delivered by Optilux 501 or when the 1 mm thick composites were exposed to light emitted by Bluephase G2, which in turn promoted a lower DC when 18 J/cm^2^ (13 s) were delivered to the 2 mm thick specimens. Radii Plus promoted DC and Rp^max^ values close to zero under most conditions, while the delivery of 56 J/cm^2^ (40 s) resulted in low DC values.

**Conclusions:**

The third generation LCU provided an optimal polymerization of Lucirin TPO-based RC under most tested conditions, whereas the second generation LED-curing unit was useless regardless of the RE.

## Introduction

In an attempt to meet patients’ growing needs for aesthetic satisfaction, clinicians are now frequently using teeth whitening techniques with their patients. As a result, bleached teeth have recently been lighter than usual^6^. In order to keep up with this new trend, manufacturers have developed resin composites (RCs) with relatively unsaturated shades and a translucency to match bleached teeth. Given that camphorquinone (CQ), the most common photoinitiator used in light-cured RCs, has a bright yellow pigment that can interfere with the resin’s color when lighter shades are required^5^ ^,^ ^27^, lighter alternatives such as 1-Phenyl 1-2propanedione (PPD) or Lucirin TPO are being added to composite formulations^9^ ^,^ ^18^.

In contrast to camphorquinone, which has an absorption peak close to 470 nm, Lucirin TPO’s absorption peak is close to 390 nm. As a consequence, ultraviolet light with a wavelength ranging between 340 nm and 430 nm is required to activate this photoinitiator^12^ ^,^ ^29^. The first and second generations of light emitting diode (LED) light curing units (LCUs) used in dentistry emit blue light in a narrow wavelength between 410 and 470 nm. Thus, they are unable to properly cure resin materials in which the CQ has been partially replaced with alternative photoinitiators^9^ ^,^ ^23^ ^,^ ^30^.

Due to the limitations of the first and second generation LED LCUs, a “third generation” was developed. Also known as “polywave”, or multi-peak LCUs, these devices emit light with a wavelength ranging between 380 and 515 nm (from ultraviolet to blue) unlike the second generation LED LCUs, which emit light with a narrower wavelength (410 – 470 nm). For this reason, these polywave LED LCUs are able to provide optimal curing in photoactivated, resin-based materials that contain only alternative photoinitiators^13^ ^,^ ^19^ ^,^ ^23^. However, because the diodes that emit ultraviolet light are not uniformly distributed on the LCU tip, polywave LED LCUs emit light of varying wavelengths through the LCU tip so that the wavelength of the beam of light is not uniformly distributed^11^. Therefore, the use of such devices can impair polymerization in some areas of the surface and on bottom layers of these RCs^14^. In this regard, it is possible that clinicians would expect that these LCUs are not as effective as quartz tungsten halogen (QTH) LCUs when different RE values are delivered to a Lucirin-based RC layer. However, there is no evidence that polywave LED LCUs are more effective than QTH light in ensuring optimal polymerization when varying RE values are applied to composite resins that contain only Lucirin TPO. In addition, clinicians could also presume that exposing Lucirin-based resin composites to light emitted by second generation LED LCUs for longer periods of time could somehow promote optimal polymerization. However, although some information is available in the literature regarding the influence of varying radiant emittance and exposure periods on the monomer conversion of RCs containing alternative photoinitiators^7^ ^,^ ^25^, no information is available about the effects of the increased exposure period of such products to curing light when using second generation LED LCUs.

This study aimed to evaluate the influence of the RE and LCU type on the degree of conversion (DC) and maximum rate of cure (Rp^max^) of experimental composite resin containing only Lucirin TPO. The following null hypotheses were evaluated. (1) Varying RE values have no influence on the DC and Rp^max^ values regardless of the LCU and RC thickness; (2) No differences in DC and Rp^max^ are noted when the Lucirin-based composite is exposed to second and third generation LEDs, or QTH LCUs regardless of the RE values delivered and thickness.

## Material and methods

### Specimen preparation

An experimental composite that contained only Lucirin TPO as a photoinitiator (Ivoclar Vivadent; Schaan, Liechtenstein) was evaluated. The RC composition is displayed in Table 1. Polyvinyl siloxane molds (Panasil, Kettenbach GmbH & Co.; Eschenburg, Germany) were made to create the square composite specimens (internal dimensions: 2 mm X 2 mm, 1 or 2 mm thick; external dimensions: 2 cm X 2 cm).

**Table 1:** Composition of experimental resin composite (provided by the manufacturer)

Componet	wt%
Urethane dimethacrylate, Bis-GMA	15.0
Ethoxylated Bis-EMA	3.8
Barium glass, ytterbium trifluoride, mixed oxide, silicon dioxide	63.5
Prepolymers	17.0
Additives, stabilizers, catalysts, pigments	0.7

Abbreviations:

Bis-GMA: Bisphenol A Glycidyl Methacrylate;

Bis-EMA: Ethoxylated Bis phenol A Dimethacrylate

The mold was positioned on the diamond surface of an attenuated total reflectance unit (ATR, Standard Golden Gate, Specac; Woodstock, GA, USA) coupled to an infrared spectroscopy unit (Tensor 27, Bruker Optik GmbH; Ettlingen, Germany). The composite was applied to the mold so that the resin bottom was in contact with the diamond surface of the ATR table. Care was taken to avoid that any pressure applied during RC’s placement into the mold deformed the mold and compromised the accuracy of the specimens’ dimensions, so the resin layer was inserted into the mold with a light pressure. The specimens were exposed to light emitted by a QTH LCU with a radiant emittance of 450 mW/cm^2^ (Optilux 501, SDS Kerr; Danbury, CA, USA); a second generation LED LCU (Radii Plus, SDI; Bayswater, Victoria, Australia) with a radiant emittance of 1400 mW/cm^2^; or a polywave LED LCU (Bluephase G2, Ivoclar Vivadent; Schaan, Liechtenstein) with a radiant emittance of 1400 mW/cm^2^. Varying exposure periods (Table 2) were selected to provide the following total RE values: 18 J/cm^2^, 36 J/cm^2^, and 56 J/cm^2^. The radiant emittance values were measured using a radiometer (Cure Rite, Dentsply Caulk; Milford, DE, USA) in order to simulate clinicians’ routines. All procedures were performed at room temperature (approximately 25°C).

**Table 2:** LCUs, radiant exposure values, radiant emittance, and exposure period evaluated in the study

LCU	Radiant exposure (J/cm2)	Radiant Emittance (mW/cm2)	Exposure period (seconds)
QTH (Optilux 501)	18	450	40
	36	450	80
	56	450	124
Second generation LED (Radii Plus)	18	1400	13
	36	1400	26
	56	1400	40
Third generation LED (Bluephase G2)	18	1400	13
	36	1400	26
	56	1400	40

Radiant emittance values obtained with a radiometer (Cure Rite, DentsplyCaulk, Milford, DE, USA)

### Degree of conversion

Real-time FTIR spectra between 1500 and 1680 cm^-1^ were obtained at a rate of 1 spectrum/second (16 scans *per* spectrum) with a resolution of 4 cm^-1^ immediately before, during and 10 min after exposure to curing light. Data were counted from the moment the infrared scan demonstrated that the resin was stabilized on the ATR surface. Based on previous studies, seven repetitions were used for each experimental group (n=7)^1^ ^-^ ^3^. Monomer conversion was calculated using standard methods that evaluated changes in the ratios of aliphatic-to-aromatic C=C infrared absorption peaks (1636 cm^-1^/1608 cm^-1^) in the uncured and cured states^21^ ^,^ ^22^ according to the following equation:


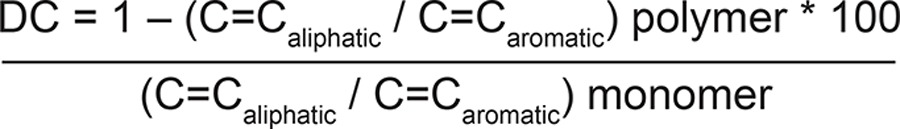


DC = 1-(C=C_aliphatic_ / C=C_aromatic_) polymer * 100

 (C=C_aliphatic_ / C=C_aromatic_) monomer 

Rp^max^ values corresponded to the highest rate of polymerization (percentage) and were calculated based on the differences between DC values measured in sequential, 1 second intervals throughout the 10 min analysis of each specimen.

The DC (%) and Rp^max^ (%/s) data were subjected to a 3-way ANOVA followed by Tukey’s *post-hoc* test (α=5%). All statistical tests and *post-hoc* power analyses were performed using a personal statistical pack (Statistics 19, SPSS Inc., IBM Company; Armonk, NY, USA).

## Results

The DC and RP^max^ results are displayed in Tables 3 and 4, respectively. The observed power of the statistical tests was over 95%. According to the 3-way ANOVA analysis and Tukey’s *post-hoc* test, a significant interaction between the “LCU,” “radiant exposure,”, and “specimen thickness” factors was observed (p<0.0001).

**Table 3:** DC means (%) (standard deviation) of Lucirin-based RCs exposed to light from different curing units and delivered radiant exposure values

Composite layer thickness: 1mm			
	18 J/cm^**2**^	36 J/cm^**2**^	56 J/cm^**2**^
Optilux 501	61.7 (1.2) ^Aa^	61.0 (1.6) ^Aa^	61.2 (3.8) ^Aa^
Bluephase G2	59.3 (2.4) ^Aa^	60.3 (1.9) ^Aa^	60.0 (2.6) ^Aa^
Radii Plus	0.6 (0.5) ^Bb^	1.1 (0.9) ^Bb^	23.2 (3.2) ^Ba^
			

**Composite layer thickness: 2 mm**			

	**18 J/cm** ^**2**^	**36 J/cm** ^**2**^	**56 J/cm** ^**2**^

Optilux 501	58.9 (2.9) ^Aa*^	61.3 (1.8) ^Aa^	59.8 (2.5) ^Aa^
Bluephase G2	51.0 (2.5) ^Bb*^	56.7 (3.2) ^Ba*^	58.8 (3.6) ^Aa^
Radii Plus	1.1 (0.9) ^Ca^	2.5 (3.7) ^Ca^	1.4 (1.6) ^Ba*^

Means followed by different letters (lower case - within row; upper case - within column) are significantly different for each composite layer thickness. Differences in DC values between RC thickness is followed by asterisk (p=0.05)

**Table 4:** Rpmax means (%) (standard deviation) of Lucirin-based RCs exposed to light from different curing units and delivered radiant exposure values

Composite layer thickness: 1 mm			
	18 J/cm^**2**^	36 J/cm^**2**^	56 J/cm^**2**^
Optilux 501	14.3 (2.0) ^Aa^	12.2 (3.4) ^Aa^	11.4 (2.8) ^Ba^
Bluephase G2	14.9 (2.4) ^Aa^	12.0 (1.5) ^Aa^	13.2 (2.0) ^Aa^
Radii Plus	0.1 (0.1) ^Ba^	0.1 (0.1) ^Ba^	4.1 (2.1) ^Cb^
			

**Composite layer thickness: 2 mm**			

	**18 J/cm** ^**2**^	**36 J/cm** ^**2**^	**56 J/cm** ^**2**^

Optilux 501	9.4 (1.9) ^Aa*^	9.2 (0.9) ^Aa*^	8.4 (1.6) ^Ba*^
Bluephase G2	9.4 (1.4) ^Aa*^	9.7 (1.3) ^Aa*^	11.0 (2.1) ^Aa*^
Radii Plus	0.1 (0.1) ^Ba^	0.1 (0.1) ^Ba^	0.1 (0.1) ^Ca*^

Means followed by different letters (lower case - within row; upper case - within column) are significantly different for each composite layer thickness. Differences in DC values between RC thickness is followed by asterisk (p=0.05)

When light from Optilux 501 or Bluephase G2 reached the 1 mm thick resin layer at varying RE values, no significant differences in DC and RP^max^ values were noted among the 18, 36, and 56 J/cm^2^ groups. Similar results were found for the 36 J/cm^2^ and 56 J/cm^2^ groups when 2 mm thick resin layers were exposed to light emitted from these two LCUs. However, when Bluephase G2 delivered 18 J/cm^2^, the DC values were lower than those obtained when higher RE values were delivered (p<0.001).

Regardless of resin thickness, exposure to Radii Plus light resulted in DC and Rp^max^ values close to zero when 18 J/cm^2^ and 36 J/cm^2^ were delivered to the RC. When 56 J/cm^2^ (40 seconds) was delivered to a 1 mm thick resin layer, the DC and Rp^max^ values were higher than those that obtained after 18 or 36 J/cm^2^, which were delivered to the resin layer (p<0.001). However, the delivery of 56 J/cm^2^ (40 seconds) resulted in significantly lower DC values than those observed when Optilux 501 and Bluephase G2 were used (p<0.001).

**Figure 1:**
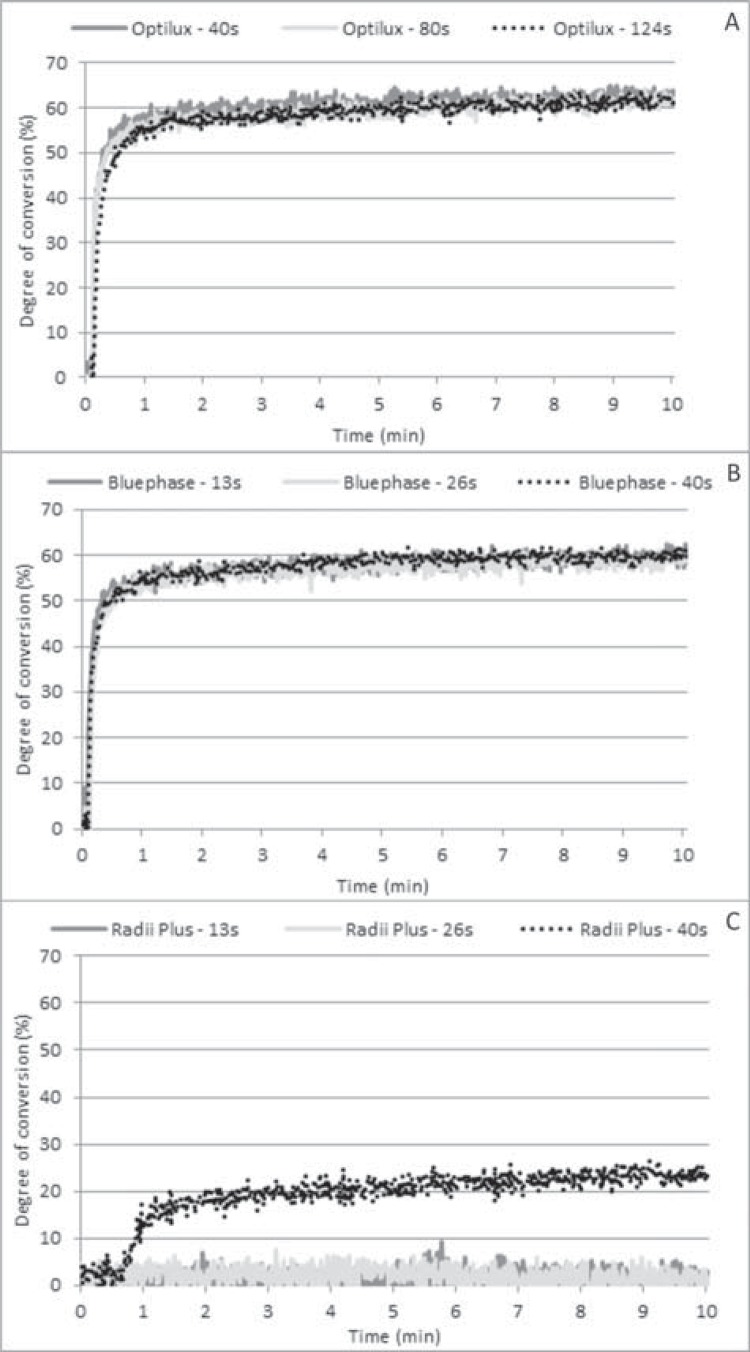
Time-based conversion of 1 mm thick RC layers exposed to Optilux 501 (A), Bluephase G2 (B), and Radii Plus (C) at varying RE values

**Figure 2:**
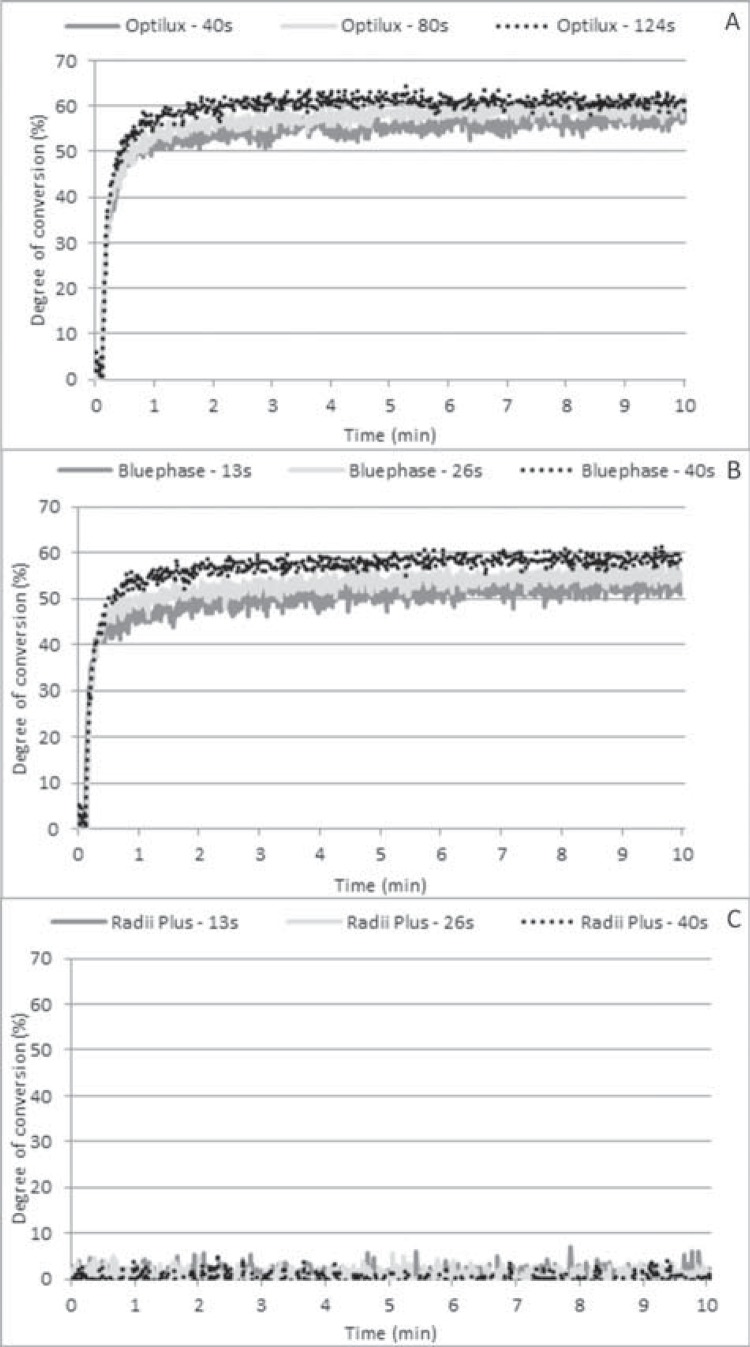
Time-based conversion of 2 mm thick RC layers exposed to Optilux 501 (A), Bluephase G2 (B), and Radii Plus (C) at varying RE values

Figures 1 and 2 show the time-based conversion profile when different RE values were delivered to 1 mm and 2 mm thick RC layers, respectively, using the tested LCUs. Optilux 501 and Bluephase G2 promoted similar time-based conversion profiles when the curing light was applied to a 1 mm thick resin layer (Figures 1A and aB). There was no polymerization when Radii Plus delivered the lowest RE values (Figure 1C). Little monomer conversion was observed when the LCU delivered the highest RE values (Figure 1C).

The time-based conversion profiles of the 2 mm thick RC layers were similar for all delivered RE values when Optilux 501 was used (Figure 2A). On the other hand, a reduction in the polymerization rate until the monomer conversion reached a plateau was observed at lower DC values when Bluephase G2 delivered lower RE values in comparison to the conversion profile observed when higher RE values were delivered (Figure 2B). As a consequence, lower 10 min DC values were noted. No polymerization was observed when Radii Plus was used regardless of the RE value (Figure 2C).

## Discussion

In the present study, the increase in the delivered RE values did not result in higher DC or Rp^max^ under most of the evaluated conditions. The only exception occurred when Bluephase G2 delivered 18 J/cm^2^ to a 2 mm thick layer, which exhibited significantly lower DC values than those of the other groups. Thus, the first hypothesis was rejected. The amount of RE delivered to the bottom of the RC layer may be influenced by factors such as photoinitiator reactivity, light scattering within the composite layer and a lack of uniformity in the light beam emitted by the LCU. In this regard, it should be noted that 17% of the light emitted by the polywave Bluephase G2 is close to the 405 nm^27^, which is required to activate Lucirin TPO. On the other hand, approximately 25% of the light emitted by Optilux 501 is in this wavelength^19^ ^,^ ^27^. Furthermore, since the light emitted by the polywave LED LCU evaluated in this study has non-uniform irradiance distribution^13^, it is possible that the light with a wavelength matching the Lucirin absorption wavelength had a lower intensity in the tip center due to the location of the LED chip that emits violet light^13^. As a consequence, the lower RE resulted in a lower DC when the polywave LED LCU was used. Such an assumption can be confirmed by using specific devices to measure the light beam profile, such as found with laser-grade beam profilers and laboratory-grade light meters, which are capable of precisely measuring spectral distribution and radiant emittance values of light emitted by such LCUs, respectively. Thus, further studies are required to confirm this guesstimate.

In this study, the QTH and polywave LED LCUs promoted higher DC values than the second generation LED LCU, which was unable to cure the RC regardless of the RE values delivered to the RC layer. This result corroborates the results of other studies^10^ ^,^ ^18^ ^,^ ^24^ and can be justified by the narrow wavelength (410 – 470 nm) emitted by that LCU. In other words, the curing light within this narrow wavelength was not capable of properly activating alternative photoinitiators such as Lucirin TPO, even when high RE values were delivered. Thus, the second hypothesis was rejected.

Curiously, the highest RE values delivered by the second-generation LED LCU resulted in little monomer conversion (approximately 23%) in the 1 mm thick RC layer. In this regard, it should be noted that the wavelength emitted by the second-generation LED covers a narrow part of the Lucirin absorption wavelength. Given that this photoinitiator may be able to start the curing up to 20 times faster than CQ^18^, it is possible that the use of high radiant emittance for a longer period of time may have led to the delivery of higher RE values within the narrow wavelength range capable of exciting Lucirin TPO. As a result, the RE amount delivered to the 1 mm thick resin layer was able to promote some monomer conversion (Table 1). Based on this finding, one could state that higher RE values delivered by the second generation LED LCU would promote an optimal polymerization. However, it should be emphasized that the RE values required to promote the high DC values found in the 1 mm thick RC layer would generate enough heat to cause irreversible pulp damage^4^ ^,^ ^8^ ^,^ ^28^.

In the present study, although shorter wavelengths (ultraviolet) were less capable of passing through the RC layer and promoting an optimal polymerization at the bottom of the resin^10^ ^,^ ^15^, the current findings demonstrated that when lower RE values were delivered to 2 mm thick resin layers, the QHT LCU was able to promote DC values similar to those observed when higher energy doses were delivered. Indeed, the delivery of 18 J/cm^2^ and 36 J/cm^2^ by the third-generation LED LCU resulted in DC values that were lower than those observed when 56 J/cm^2^ was delivered and when the 1 mm thick resin layers were exposed to the light emitted by such an LCU. On the other hand, in most groups, the use of a thicker resin layer resulted in Rp^max^ values that were lower than those of the 1 mm thick resin layers. However, it should be emphasized that DC values were evaluated 10 min after exposure to light. Therefore, it is possible that the RC layers that showed lower Rp^max^ values would exhibit faster post-polymerization after the light was shut off than the resin layers that showed higher Rp^max^ values. As a consequence, despite the difference in Rp^max^ values, the faster post-polymerization after polymer vitrification in resin layers with lower Rp^max^ values would compensate for the apparently lower initial monomer conversion observed right after the light was shut off. This could be noted in the time-based conversion analysis, mainly when lower RE values were delivered to the RC layer (Figures 2A and 2B). As a result, further polymerization was expected after 10 min. Thus, the DC values obtained with the 2 mm thick resin layers after 13 s of exposure can increase within the first 24 hours^20^. As a result, the DC values after longer evaluation periods could be close to, or even similar to, those observed when higher RE values were delivered. However, only further studies evaluating monomer conversion for longer periods of time can confirm this speculation.

In the present study, a regular dental radiometer was used to measure the radiant emittance of the tested LCUs so that only the total radiant emittance values were determined. For this reason, the calculated RE values were not based on specific wavelengths but on the sum of radiant emittance from all wavelengths, which is routinely used by clinicians. In addition, such hand held radiometers do not provide accurate radiant emittance values of the light emitted by LED LCUs in comparison to those provided by laboratory-grade meters^16^ ^,^ ^17^ ^,^ ^26^. Therefore, it is evident that this radiometer may not be effective in determining the proper exposure period to ensure the optimal polymerization of the RC layer.

Based on the current findings, the evaluated polywave LCU can be as effective as the QTH LCU for providing optimal polymerization of Lucirin-based RC, but only when proper RE is delivered. In this regard, clinicians should be aware of the RC composition and LCU light beam profile. With this information in hand, they will be able to determine the minimum exposure period and optimal resin layer thickness for ensuring that optimal polymerization is achieved in every RC layer. In addition, longer exposures of RC layers to light emitted from second generation LED LCUs should be avoided as they do not compensate for the differences between the Lucirin TPO absorption wavelength peak and the LCU light wavelength.

## Conclusion

Based on the current findings and within the limitations of the present study, it can be concluded that:

- The use of polywave LED LCUs can result in lower DC values when lower RE values are delivered to thicker RC layers that only contain Lucirin TPO.
- Both the polywave LED and QTH LCUs promoted higher DC and Rp^max^ values than did the second generation LED LCU regardless of the delivered RE value.
- Although no difference in the Rp^max^ values were found, thicker RC layers showed lower DC than thinner layers when the lowest RE value (18 J/cm^2^) was delivered by the polywave LED LCU.
